# Supplementary material for: Transcriptome Analysis for Salt-Responsive Genes in Two Different Alfalfa (Medicago sativa L.) Cultivars and Functional Analysis of MsHPCA1
Source: Plants (Basel). 2024 Apr 11;13(8):1073. doi: 10.3390/plants13081073 (PMC11054072; doi:10.3390/plants13081073)
Supplement: Supplementary file 1 [file plants-13-01073-s001.zip › plants-2922556-supplementary.pdf]

Table S1 Summary of read numbers based on the RNA-Seq data from two varieties alfalfa seedling.

	Sibeied			Gongnong NO.1		
	0h	12h	24h	0h	12h	24h
Total reads	23,193,268	22,249,331	23,136,572	23,360,050	23,152,698	21,849,447
Mapped reads	18,570,963	17,811,746	18,528,072	18,603,816	18,809,403	17,791,533
Unique match	17,348,603	16,556,222	17,238,635	17,335,489	17,550,090	16,555,858
Multi-position match	1,222,360	1,255,524	1,289,437	1,268,327	1,259,313	1,235,675
Unmapped reads	4,622,305	4,437,585	4,608,500	4,756,234	4,343,295	4,057,914

Table S2 DEGs involved in 'Gongnong No.1' at 12h and 24h after salt tolerant.

Gene ID	Expression	Levels	Description
	G12h	G24h	
Glycometabolism-associated			
MsG0680031838	6.72	5.27	UDP-glucosyltransferase
MsG0780038334	3.81	3.99	UDP-glucosyltransferase
MsG0580028342	3.81	3.23	UDP-glucosyltransferase
MsG0880047496	2.84	2.53	UDP-glucosyltransferase
MsG0480020934	2.64	2.73	UDP-glucosyltransferase
MsG0780038123	2.58	3.04	UDP-glucosyltransferase
MsG0880045104	2.48	2.00	UDP-glucosyltransferase
MsG0380015885	2.18	2.34	UDP-glucosyltransferase
MsG0880042173	2.09	1.12	UDP-glucosyltransferase
MsG0280011136	2.03	1.52	UDP-glucosyltransferase
MsG0780039877	1.89	1.69	UDP-glucosyltransferase
MsG0480023272	1.86	2.69	UDP-glucosyltransferase
MsG0680035715	1.77	1.57	UDP-glucosyltransferase
MsG0480018495	1.76	1.38	UDP-glucosyltransferase
MsG0380017787	1.75	2.13	UDP-glucosyltransferase
MsG0880045105	1.71	2.55	SWEET sugar transporter
MsG0880046908	1.71	1.58	Sugar/inositol transporter
MsG0380013908	1.63	1.52	Sugar phosphate transporter
MsG0480021781	1.60	1.29	Sugar phosphate transporter
MsG0180005764	1.56	2.08	Sugar transporter-like
MsG0680035710	1.57	1.10	Sucrose synthase
MsG0180001050	1.39	1.31	Glyoxalase-like
MsG0780039739	1.35	1.44	Glycosyl transferase 8
MsG0680030659	1.33	1.27	Glycosyl transferase 14
MsG0780038116	1.31	1.78	Glycosyl hydrolases 36
MsG0880046325	1.27	1.32	Glycoside hydrolase 5
MsG0280009291	1.23	1.49	Glycoside hydrolase 3
MsG0580027360	1.23	1.21	Glycoside hydrolase 28
MsG0380011504	1.16	1.14	Glycoside hydrolase 28
MsG0380016061	1.15	1.06	Glycoside hydrolase 28
MsG0780038117	1.14	1.75	Glycoside hydrolase 28
MsG0280007901	1.13	1.57	Glycoside hydrolase 27
MsG0280009307	1.07	1.38	Glycoside hydrolase 19

MsG0880043127	1.02	1.26	Glycoside hydrolase 19
MsG0280008645	−1.08	−1.08	Glycoside hydrolase 18
MsG0580030055	−1.10	−1.04	Glycoside hydrolase 17
MsG0480022515	−1.25	−1.11	Glycoside hydrolase 16
MsG0380015190	−1.34	−1.36	Glycoside hydrolase 1
MsG0180004351	−2.33	−1.98	Cellulose synthase
MsG0780040306	−3.67	−3.39	Galactose-binding-like
Energy-associated			
MsG0180005882	7.14	5.92	ATPase, F1/V1/A1 complex
MsG0180000608	1.58	2.14	ATPase, AFG1-like
MsG0380016836	1.16	1.39	AAA+ ATPase
MsG0880045924	2.44	2.59	P-type ATPase
MsG0580025389	1.24	1.19	AMP-dependent synthetase
MsG0680031843	−1.30	−1.31	AAA+ ATPase
MsG0580025521	−1.10	−2.20	Small GTPase
MsG0780038417	3.18	3.79	NAD(P)-binding
MsG0580024178	2.04	1.97	NADH/Aldolase-type TIM barrel
MsG0780041642	1.25	1.42	NADP-dependent oxidoreductase
MsG0780036964	1.74	1.15	FMN-binding split barrel
MsG0480020444	1.39	1.77	FAD/NAD(P)-binding
MsG0380011901	4.73	4.23	Oxygen oxidoreductase covalent FAD-binding site
MsG0280007749	1.09	1.47	FAD linked oxidase
MsG0880042930	−1.70	−2.11	FAD linked oxidase
MsG0480022692	4.56	6.15	Mitochondrial substrate
MsG0280011053	1.50	1.50	Mitochondrial substrate
MsG0480018326	−1.10	−1.41	Mitochondrial substrate
Transcription factor-associated			
MsG0280010800	6.85	6.40	Zinc finger, RING-type
MsG0180004800	3.09	3.04	Zinc finger, RING-CH-type
MsG0480020428	2.75	1.53	Zinc finger, RING-type
MsG0780039229	1.55	1.20	Zinc finger, RING-type
MsG0880046674	−1.25	−1.31	Zinc finger, RING-type
MsG0480023441	−2.67	−2.34	Zinc finger, RING-type
MsG0180003673	−3.73	−4.30	Zinc finger, RING-type
MsG0380012512	2.52	2.72	Zinc finger, CCHC-type
MsG0880045175	−1.17	−1.10	Zinc finger, CCHC-type
MsG0880046752	1.04	1.57	Zinc finger, CCCH-type
MsG0380017253	−1.43	−1.33	Zinc finger, CCCH-type

MsG0080048430	−1.48	−1.27	Zinc knuckle CX2CX4HX4C
MsG0880042051	6.86	6.44	AP2/ERF
MsG0580024424	6.21	5.27	AP2/ERF
MsG0380017423	3.58	2.90	AP2/ERF
MsG0180005095	−1.53	−1.15	AP2/ERF
MsG0780041742	3.02	2.23	WRKY
MsG0280011473	1.49	1.33	WRKY
MsG0380013522	2.75	2.24	SANT/Myb
MsG0780040741	2.47	2.41	SANT/Myb
MsG0280010653	2.30	1.87	SANT/Myb
MsG0480020725	1.81	2.58	SANT/Myb
MsG0380015227	1.30	1.24	SANT/Myb
MsG0180004180	−1.12	−1.59	SANT/Myb
MsG0280011041	1.29	1.53	GRAS
MsG0480023497	1.25	1.50	NAC
MsG0280009717	1.00	1.17	NAC
MsG0180001854	−2.38	−1.37	NAC
MsG0180005527	1.17	1.17	bHLH
MsG0580025575	1.06	1.42	bHLH
MsG0280010124	−1.14	−1.30	TCP
Stress-associated			
MsG0780040578	7.41	7.36	Late embryogenesis abundant protein 25
MsG0580028758	2.21	1.74	Late embryogenesis abundant protein 14
MsG0580027808	2.10	1.88	Late embryogenesis abundant protein14
MsG0580028668	1.89	2.08	Late embryogenesis abundant protein14
MsG0180000472	5.99	5.71	Hsp20-like
MsG0480022345	3.69	4.27	Hsp20
MsG0480022532	3.67	4.59	Hsp20
MsG0480022344	3.15	4.50	Hsp20
MsG0280008285	1.78	1.41	Hsp70
MsG0880045617	1.44	1.80	Hsp90-like
MsG0680031459	5.24	3.07	EF-hand
MsG0880046234	4.26	3.17	EF-hand
MsG0180000257	2.66	3.37	EF-hand
MsG0880047171	1.41	1.21	EF-hand
MsG0380017134	1.72	1.81	EF-hand
MsG0780038940	3.36	2.49	Glutathione S-transferase
MsG0180004947	2.51	2.18	Glutathione S-transferase

MsG0780038939	2.34	2.09	Glutathione S-transferase
MsG0280009765	2.18	2.45	Glutathione S-transferase
MsG0680030336	2.18	1.76	Thioredoxin-like ferredoxin
MsG0180004941	2.00	2.02	Glutathione S-transferase
MsG0180004879	1.82	1.64	Glutathione S-transferase
MsG0380012122	1.61	1.61	Thioredoxin
MsG0580026027	1.41	1.97	Thioredoxin
MsG0380011844	1.35	1.07	Thioredoxin
MsG0180004880	1.34	1.63	Glutathione S-transferase
MsG0780039805	1.18	1.28	Glutaredoxin
MsG0180004942	1.07	1.29	Glutathione S-transferase
MsG0180004895	1.08	1.34	Glutaredoxin
MsG0580027201	2.25	2.71	Plant peroxidase
MsG0380016748	2.23	1.51	Plant peroxidase
MsG0880045686	1.70	1.50	Plant peroxidase
MsG0880045955	1.39	1.16	Plant peroxidase
MsG0880045973	1.37	1.35	Plant peroxidase
MsG0280007687	1.22	1.45	Plant peroxidase
MsG0580029291	1.03	1.60	Plant peroxidase
MsG0480018530	1.70	1.74	Toll/interleukin-1 receptor homology (TIR)
MsG0880044837	1.62	1.38	Toll/interleukin-1 receptor homology (TIR)
MsG0480022752	1.33	1.22	Stress up-regulated Nod 19
MsG0380015868	1.05	1.13	Manganese/iron superoxide dismutase

Table S3 DEGs involved in ' Sibeide' at 12h and 24h after salt-tolerant.

Gene ID	Expression Levels		Description
	S12h	S24h	
Glyco-metabolism-associated			
MsG0780039323	3.00	3.37	UDP-glucosyltransferase
MsG0880041992	−1.28	−1.58	UDP-glycosyltransferase
MsG0180005810	−1.42	−1.25	UDP-glycosyltransferase
MsG0680030477	1.38	1.48	SWEET sugar transporter
MsG0680030470	1.30	1.57	SWEET sugar transporter
MsG0280010202	−1.68	−1.85	Sugar phosphate transporter
MsG0680030621	−1.74	−1.10	Glycosyl transferase 2-like
MsG0280008254	1.53	1.04	Glycosyl transferase 14
MsG0780040702	1.03	1.71	Glycosyl transferase 8
MsG0580025982	−1.10	−1.05	Glycosyl transferase 92
MsG0580027605	2.05	1.41	Glycoside hydrolase
MsG0480022184	1.85	1.92	Glycoside hydrolase
MsG0180004349	1.67	1.42	Glycoside hydrolase 27/36
MsG0880047761	1.65	1.48	Glycoside hydrolase 17
MsG0880047207	1.39	1.09	Glycoside hydrolase 1
MsG0480021114	1.20	1.26	Glycoside hydrolase 1
MsG0180000436	−1.07	−1.55	Glycoside hydrolase 32
MsG0180000437	−1.39	−1.57	Glycoside hydrolase 32
MsG0480021131	−1.66	−2.08	Glycoside hydrolase 1
MsG0880041896	−1.36	−1.77	Malate dehydrogenase, type 2
MsG0680034483	1.38	1.52	Fructose-bisphosphate aldolas
MsG0780041081	1.37	1.58	Fructose-bisphosphate aldolase
MsG0480021327	1.37	2.37	Fructose-bisphosphate aldolase
MsG0780041082	1.17	1.38	Fructose-bisphosphate aldolase
MsG0080048965	2.83	2.52	Trehalose-phosphatase
MsG0180000567	−1.23	−1.36	Chitinase II
MsG0680030406	−1.59	−1.18	Pectin lyase
Energy-associated			
MsG0780036265	2.27	2.38	AAA+ ATPase
MsG0380013942	1.63	1.86	ATPase, F1/V1/A1 complex
MsG0380017406	1.40	1.57	ATP synthase, F0 complex, subunit
MsG0880046219	1.12	1.26	ATP synthase OSCP
MsG0780041367	1.00	1.20	ATP-dependent Clp protease proteolytic subunit
MsG0380015758	−1.02	−1.09	ATPase, AAA-type
MsG0880042935	−1.11	−1.18	Putative ATP-synthase-associated protein
MsG0380016571	−1.15	−1.47	ATPase, AAA-type

MsG0180003504	−1.59	−1.24	AMP-dependent synthetase enzyme
MsG0780041383	−2.26	−1.42	AMP-dependent synthetase
MsG0780039304	6.42	4.73	NADP-dependent oxidoreductase
MsG0380017196	1.15	1.51	NAD(P)-binding
MsG0280007079	1.15	1.00	NAD(P)-binding
MsG0180003730	−1.17	−1.00	NAD(P)-binding
MsG0580025511	−1.24	−1.30	NAD(P)-binding
MsG0680030421	−1.60	−1.96	NAD(P)-binding
MsG0780039576	−1.78	−1.44	NAD-dependent epimerase/dehydratase
MsG0580024990	−1.80	−1.06	NAD(P)-binding
MsG0480020487	−1.86	−1.15	NAD(P)-binding
MsG0580030133	−2.00	−1.23	NADP-dependent oxidoreductase
MsG0580030135	−2.09	−1.23	NADP-dependent oxidoreductase
MsG0480021361	−2.67	−2.77	NADP-dependent oxidoreductase
MsG0280011016	−3.91	−3.29	NAD-dependent epimerase/dehydratase
MsG0180002255	−1.33	−1.10	NADPH-dependent FMN reductase-like
MsG0880044675	−2.62	−2.41	NADPH-dependent FMN reductase-like
MsG0880044676	−3.06	−2.22	NADPH-dependent FMN reductase-like
MsG0480018405	1.04	1.07	Cryptochrome/DNA photolyase, FAD-binding
MsG0880043920	−1.38	−1.19	Oxidoreductase FAD/NAD(P)-binding
MsG0880046296	−1.64	−1.78	FAD-binding 8
Transcription factor-associated			
MsG0580028462	1.82	1.55	Zinc finger, RING-type
MsG0180003687	−1.10	−1.63	Zinc finger, RING-type
MsG0880046215	−1.15	−1.99	Zinc finger, RING-type
MsG0480023693	−1.21	−1.16	Zinc finger, RING-type
MsG0080047905	−1.24	−1.22	Zinc finger, RING-type
MsG0180003595	−1.28	−1.27	Zinc finger, RING-type
MsG0880043098	−1.32	−1.40	Zinc finger, RING-type
MsG0580028016	−1.21	−1.02	Zinc finger, CCCH-type
MsG0580026614	−1.31	−1.05	Zinc finger, CCHC-type
MsG0580026192	−2.98	−3.58	Zinc finger, Dof-type
MsG0380015805	−1.14	−1.54	Zinc finger, Dof-type
MsG0580029912	1.37	1.93	zinc ribbon
MsG0780041106	1.29	1.40	Zinc finger, AN1-type
MsG0580027325	1.04	1.04	Basic-leucine zipper
MsG0280010515	−1.02	−1.06	Basic-leucine zipper
MsG0480023067	−1.43	−1.50	Basic-leucine zipper
MsG0880046457	−1.13	−1.25	ZF-HD homeobox
MsG0180003830	−2.03	−1.75	AP2/ERF
MsG0580029966	1.72	1.59	CBF

MsG0380016957	1.33	1.33	CBF
MsG0280008749	3.64	3.77	MADS-box
MsG0180000525	2.42	2.17	WRKY
MsG0280007786	−1.51	−2.43	WRKY
MsG0080048036	−1.68	−2.19	WRKY
MsG0380014706	−1.13	−1.89	SANT/Myb
MsG0580024732	−3.93	−4.68	SANT/Myb
MsG0280011042	−1.26	−1.75	GRAS
MsG0680032758	−1.05	−1.08	GRAS
MsG0580024328	−1.41	−1.55	GRAS
MsG0580025534	−1.10	−1.32	bHLH
MsG0880043481	−2.03	−1.80	bHLH
MsG0480022288	−2.30	−1.66	bHLH
MsG0280010611	−1.38	−1.34	Homeobox
MsG0480019666	−1.73	−1.11	K-box
Stress-associated			
MsG0180000047	1.32	1.40	Hsp90
MsG0380015992	1.20	1.47	Hsp70
MsG0280006520	1.00	1.26	Hsp70
MsG0780040480	−1.03	−1.34	Hsp90−type
MsG0480022400	−2.29	−2.58	Hsp−type
MsG0780038722	1.63	1.14	EF−hand
MsG0880043002	−1.09	−1.10	EF−hand
MsG0280011105	−1.09	−1.75	EF−hand
MsG0880043460	−1.39	−1.82	EF−hand
MsG0880047006	2.17	2.40	Glutaredoxin
MsG0880045094	1.47	1.11	Thioredoxin−like
MsG0580026148	−1.50	−1.30	Glutathione S−transferase
MsG0680030740	−1.92	−1.72	Glutaredoxin
MsG0580025023	−1.20	−1.10	Plant peroxidase
MsG0780039390	2.10	2.33	Vitamin C permease
MsG0380016734	1.03	1.29	Manganese/iron superoxide dismutase

Table S4 Primer sequence associated with this article experiments.

Gene	Forward primer/ Reverse primer（5′−3′）	Amplicon length (bp)
MsHPCA1−ORF−F	ATGGGTGAAAGAACTCTAGTGTTCCTACTCTTCCTTTTCAG	2865
MsHPCA1−ORF−R	TTATGCACGCGGAAGTATCACACTTGAATCAAAGTACTC	
MsHPCA1−pENTER−tasiRNA−F	GGAATTCAAGAACTGGAACATTCTGGAATACCTTGCACTTTCAACTTTAAAG	300
MsHPCA1−pENTER−tasiRNA−R	CGGGATCCACCAGTGAAACCACAATTAATAAG	
MsHPCA1−pQB−V3−F	CTTTGTACAAAAAAGCAGGCTCAGGGGATGATATCATGGGTGAAAGAACTCTAGTG	2856
MsHPCA1−pQB−V3−R	TTGTACAAGAAAGCTGGGTGCAGGGCGATGATATCTGCACGCGGAAGTATC	
MsHPCA1−pENTER−OE−F	GTCGACGGTATCGATAAGCTTATGGGTGAAAGAACTCTAGTG	2856
MsHPCA1−pENTER−OE−R	CCAAATGTTTGAACGTCTAGATGCACGCGGAAGTATC	
MsHPCA1−qpcr−F	ATGATTCAGTAATAGAAGGTCCTGT	198
MsHPCA1−qpcr−R	TGCTCTCATCAGGACCCCA	
MsActin−qpcr−F	GCTGACCGTATGAGCAAGGA	114
MsActin−qpcr−R	TGCCAAGATAGACCCACCAA	
